# Effect of Early Intervention on Language Development in Hearing-Impaired Children 

**Published:** 2016-01

**Authors:** Elahe Shojaei, Zahra Jafari, Maryam Gholami

**Affiliations:** 1 *Department of Audiology, Rehabilitation Sciences Faculty, Iran University of Medicine Sciences, Tehran, Iran. *; 2 *Department of Basic Sciences in Rehabilitation, Rehabilitation Sciences Faculty, Iran University of Medical Sciences, Tehran, Iran.*; 3 *Clinical speech therapist, Social Welfare Organization, Tehran, Iran.*

**Keywords:** Child, Early identification, Early intervention, Hearing loss, Language development

## Abstract

**Introduction:**

Hearing loss from birth up to the age of 3 years has a negative effect on speech/language development and results in sensory, cognitive, emotional, and academic defects in adulthood by causing delayed development of communicative-linguistic abilities. The present study was performed in order to assess the effect of early intervention on language development in Persian children aged 6-7 years with severe sensorineural hearing loss.

**Materials and Methods:**

Thirty boys and girls aged 6-7 years participated in this study, all of them had severe congenital sensorineural hearing loss in both ears. All children were using bilateral behind-the-ear hearing aid, and had similar economic/socio-cultural backgrounds. Subjects were categorized into two groups based on the age of identification/intervention of hearing loss (3-6 and 12-15 months of age). The Persian TOLD-P3 test was used to evaluate language development in all subjects. Data collection was accomplished by observation, completion of questionnaires, and speech recording.

**Results:**

There was a significant difference in language development in 11 sub-tests and five lingual gains on the Persian TOLD-P3 test between early (3-6 months of age) and late identified/intervened (12-15 months of age) hearing-impaired children (P<0.05). Early identified/intervened hearing-impaired children had a notable preference in all assessed sub-tests and lingual gains.

**Conclusion:**

Early identification/intervention of hearing loss before the age of 6 months has a significant positive effect on a child’s language development in terms of picture/relational/oral vocabulary, grammatical comprehension, sentence combining, grammatical completion, phonologic analysis, word differentiation, word production, semantics, and syntax. Moreover, early identification/ intervention of hearing loss develops the hearing-impaired child’s lingual gains in visual vocabulary, grammatical completion, word differentiation, phonologic analysis, and word production.

## Introduction

Verbal language perception, development, and usage is strongly related to the auditory sense. Therefore, the presence of hearing loss – even to a mild extent – has a negative effect on speech–language development in hearing-impaired children, and delays acquisition of linguistic, social, academic, and sensory abilities. Further, as speech and language development are prerequisites for cognitive development, an auditory defect may affect and impair the hearing-impaired child’s cognitive ability (1,2). Verbal language is a humanized skill which is acquired gradually during a defined step-by-step process. Language is acquired through daily life interactions without any training in normal-hearing children. Hearing loss hampers this process and causes language disorder. Therefore, normal language acquisition in the hearing-impaired child requires special training based on the degree of hearing loss (3-5).

Pre-lingual hearing loss has a negative effect on all fields of language acquisition, but the influence on phonology,morphology, advanced vocabulary, and syntax is most profound (6). Because of the dramatic decreased hearing sensitivity in moderately severe or severe hearing loss, delay of speech and language development in hearing-impaired children is not unexpected. Since full compensation of auditory defects is not possible solely by acoustic amplification, lip/speech reading and even sign language training in some cases is needed for normal cognitive development in hearing-impaired children (7). The first 36 months of childhood are the most critical periods in terms of language acquisition, and language development is never again as rapid after this period (8,9). Reception and perception of acoustic stimuli are essential prerequisites for pre-lingual activities. Therefore, early hearing-loss identification accompanied by appropriate intervention is essential for normal language acquisition in hearing-impaired children (10,11).

Identification of hearing loss and early appropriate intervention before the age of 6 months can increase the possibility of normal speech and language development in hearing-impaired children (12,13). The appropriate intervention program must include family consultation, hearing aid description/fitting, auditory training, language learning, and educational strategies based on the needs and abilities of the baby or child (14). Early identification and intervention are the variables with the greatest impact on speech and language development. Other important variables are degree of hearing loss, intelligence quotient (IQ), other disabilities, socio-familial/ cultural background, family communicative pattern, gender, and the mother’s level of literacy (15,16). The parent’s hearing sensitivity and their method of communication – verbal or sign language – also have indirect effects on the hearing-impaired child’s communicative abilities. Late identification/intervention of hearing loss results in development of a restricted vocabulary, grammatical problems and academic difficulties (17). Moreover, hearing-impaired children use shorter and simpler sentences than children with normal hearing, consisting of names and verbs only. These children seldom use functional words in their sentences. Studying the language abilities of hearing-impaired children requires the use of a precise method for evaluating both expressive and perceptive language at each age level (18).

Many studies have confirmed the significant positive effects of early identification of hearing loss on speech, language, and socio-emotional develop- ment. Murria et al. showed that hearing-impaired children who have received appropriate and early hearing aid assessment and fitting at the age of 3 months and cochlear implantation at the age of 9 months can reach normal language development in up to 96% of cases (19). Hearing-impaired children who have received early identification/intervention in the very first 2 months of life (or at the age of 3–4 or 5–6 months) have similar language development. This means that early and appropriate identification/ intervention of hearing loss before the age of 6 months enables normal language development in hearing-impaired children (20). By comparing early identification/ intervention (3-4 or 5-6 months of age) with late hearing loss (7-12,13-18,19-24, or 25-30 months of age), a considerable improvement in language development is revealed in those children identified early (20). 

The presence of hearing loss at critical periods of language development causes disorders in speech acoustic processing and language synthetics–syntax representation and results in defects in language acquisition and synthetics-syntax usage. Language learning in hearing-impaired children requires the presence of natural conditions; therefore, their lingual environment must be the same as for normal-hearing children. Most synthetic and syntax abilities are learned at critical periods of language development, and this is affected by different variables such as the mother’s speech, the complexity of heard sentences and repetition–communicative situations (21). Despite making progress in reducing the age of identification/intervention in hearing-impaired children, language development gaps between normal-hearing and hearing-impaired children still exist. Decreasing these gaps will allow more opportunities for evaluating the lingual abilities and rehabilitation program planning of hearing-impaired children based on their needs and abilities (22,23).

It is note-worthy that the development of semantic and syntax skills are the basis of academic progress in school. As school entrance age is 6–7 years in Iran, evaluating these skills before entering the school will be useful for educational/ verbal rehabilitative program planning. Despite the availability of several similar studies concerning verbal skills in Persian hearing-impaired children, there are currently no published studies on verbal skills in Persian children aged 6–7 years with hearing impairment. However, suitable rehabilitative program planning requires accurate identification of language deficiency for every hearing-impaired child, individually. This may be obtained through precise and detailed evaluation of hearing-impaired abilities in different semantic and syntax aspects by utilizing a proper and plenary tool such as TOLD-P3. Although several studies have been published relating to the effect of early intervention on language development in Persian hearing-impaired children, none have used such a test. Hence, our study is the first research into the evaluation of lingual gains in Persian hearing-impaired children, and is thus an unprecedented and innovative study of the lingual abilities of Persian children with hearing impairment. Considering the importance of language development as a principal prerequisite for socio-academic success at school, this study was performed to determine the effect of early hearing loss intervention on language development in Persian children aged 6–7 years with severe sensorineural hearing impairment before starting school.

## Materials and Methods

Thirty children (14 girls and 16 boys) aged 6–7 years with severe sensorineural hearing impairment participated in our study. The entry criteria were having bilateral congenital flat severe sensorineural hearing loss (70–85dB), normal tympanic membrane, tympanogram and IQ scores (based on Goodenough–Harris Test scores of 90–110), no other handicap, binaural hearing aid fitting (used for 12–14 hours per day), and the similarity of the intervention program received. All children had the same socio-cultural background and were classified into two groups depending on the age of hearing loss identification/ intervention. The first group consisted of six girls and nine boys with a hearing-loss identification/ intervention age of 3–6 months and the second group consisted of seven girls and eight boys with a hearing-loss identification/intervention age of 12–15 months. These children were selected among other children supported by the Narmak Welfare Organization Center by an available sampling method. All subjects were monolingual, right-handed children who lived with literate, normal-hearing parents. Their communicative pattern was verbal language. Safety and ethical aspects of this research project were ensured by the Iran Medicine Sciences University.

The first stage of the data collection process was completion of a questionnaire consisting of questions about the individuals' medical-familiar history, age of hearing loss identification/ intervention, quality of intervention program (auditory training, speech reading, and lip reading). Next, an audiologic evaluation including otoscopy, immittance and pure tone audiometry was performed in all children. Immittance and pure tone audiometry were undertaken in the Narmak Center's audiology clinic using a Pejvak Ava ZA86 and Pejvak Ava CA86 clinical audiometer, respectively. Acoustic stimuli were delivered via TDH39 supra-aural headphones. The lingual abilities were assessed using the Persian TOLD-P3 test as well as direct observation, questionnaire completion and speech recording as performed at the end of the data collection process (24). Raw and standardized scores were calculated for each of the sub-tests and lingual gains. TOLD-P3 is one of the most comprehensive lingual tests containing 11 sub-tests: pictures/ relational/ oral vocabulary, grammatical comprehe- nsion, sentence combining, grammatical completion, phoneme analysis, word differentiation, word production, and semantics and syntax. Combining the 11 sub-tests gives five lingual gains: visual vocabulary, grammatical completion, word differentiation, phoneme analysis, and word production (24). Interpretation of standardized scores for all sub-tests and lingual gains was performed based on the criteria in (Table. 1).

**Table1:** The interpretation criteria of standard scores on TOLD-P3 sub-tests and lingual gains

**Description**	**Lingual gain(standard score)**	**Description**	**Sub-test(standard score)**
Very excellent	>121	Very excellent	17-20
excellent	121-130	excellent	15-16
Above than moderate	111-120	Above than moderate	13-14
moderate	90-110	moderate	8-13
Lower than moderate	80-89	Lower than moderate	6-7
fair	70-79	Poor	4-5
Very poor	<69	Very poor	1-3

The Kolmogorov–Smirnov test showed normal distribution of the sample. An independent T–test was used to compare the means of scores in all sub-tests and lingual gains.

SPSS18 used to perform statistical analysis, and the putative level of significance was defined as P<0.05.

## Results

Descriptive data relating to standardized scores for picture/ relational/oral vocabulary, grammatical comprehension, sentence combining, grammatical completion, phonologic analysis, word differentiation, word production, semantics and syntax are presented in (Table.2).

**Table2:** The mean and standard deviation of standards scores in TOLD-P3 sub-tests

**Sub-test (standard scores)**	**Standard deviation**	**mean**	**Group**	**Sub-test(standard scores)**
Picture vocabulary	first second	13.93 7.53	1.48 1.21	0.001
Relational vocabulary	first second	12.85 6.48	1.07 1.67	0.001
Oral vocabulary	first second	12.32 7.95	1.23 1.23	0.001
Grammatical comprehension	first second	12.74 8.32	1.56 1.42	0.001
Sentence combining	first second	13.01 9.14	1.71 1.65	0.001
Grammatical completion	first second	13.32 28.8	1.12 1.32	0.001
Word differentiation	first second	12.48 7.96	0.58 0.93	0.001
Phonologic analysis	first second	12.93 8.21	2.18 1.99	0.001
Word production	first second	12.14 7.95	0.85 0.97	0.001
semantics	first second	12.32 8.00	1.18 1.87	0.001
Syntax	first second	11.91 6.54	1.13 1.57	0.001

The first group: early hearing loss identified/intervened children (N=15)

Comparing the results obtained from these 11 sub-tests showed a statistically significant difference between lingual abilities in the two groups (P<0.05). Moreover, there was a significant difference in the standardized scores for the five lingual gains (visual vocabulary, grammatical completion, word differentiation, phoneme analysis and word production) between the early and late identification/intervention groups (P<0.05) (Table. 3).

**Table 3:** The mean and standard deviation of standards scores in TOLD-P3 lingual gains

**Lingual gain ** **(standard scores**	**First group Mean** **(standard deviation)**	**Second group Mean** **(standard deviation)**
Visual vocabulary	115(1.121)	82(1.41)
Grammatical completion	109(0.98)	80(1.09)
Word differentiation	111 (1.92)	85(1.63)
Phonologic analysis	109(1.44)	76(0.86)
Word production	112 (0.88)	84(1.03)

First group: early hearing loss identified/intervened children (N=15), Second group: late hearing loss identified/intervened children (N=15)

## Discussion

This study showed a significant difference between the developments of lingual abilities in children with early compared with late identified/intervened hearing loss. Lingual aspects assessed were abilities in semantics, syntax and phonology through 11 sub-tests (picture/ relational/ oral vocabulary, grammatical comprehension, sentence combining, grammatical completion, phonologic analysis, word differentiation, word production, semantics and syntax) and five lingual gains (visual vocabulary, grammatical completion, word differentiation, phoneme analysis and word production). Ability in semantic ability was evaluated by picture/relational/oral vocabulary and semantic sub-tests. Knowledge of vocabulary, objects and events constitute semantic ability, which offers the possibility of thinking about language and talking about words, as well as their proper use. Thus, the development of semantic ability provides the basis for using a defined word for a defined function or target expression (25). Hearing loss restricts the rate of development of vocabulary in hearing-impaired children in comparison with normal-hearing cases. This difference will be more pronounced in older-aged children. There is some semantic delay in all development periods in hearing-impaired children; therefore, these children often have difficulties in making long complex sentences, conceptual multiple meaning, and abstract word perception. Thus, the process of learning-based speaking and writing is weaker in hearing-impaired children than in normal-hearing children (25).

The significant difference between syntactic skills in children with early versus late identified/intervened hearing loss was another finding that was evaluated by grammatical comprehension, sentence combining and grammatical completion of sub-tests. These differences support the negative effect of late identification of hearing loss on syntactic abilities and the importance of early appropriate hearing loss intervention in hearing-impaired children. Syntactic skills enable children to use syntactic morphemes, adverbs, prepositions, pronouns, compound sentences and verb suffixes properly. Based on their degree of hearing loss and the quality of the intervention program used, hearing-impaired children have syntactic difficulties in such a way that their syntactic construction significantly relies on putting disjointed single words together. This phenomenon is not seen in the lingual construction of normal-hearing children (25). 

Improvement in phonologic skills was assessed by word production, word differentiation and phonologic analysis sub-tests in the both early and late identified/intervened hearing-loss children. There was a significant difference between phonologic skills in the two groups; early identified/intervened impaired-hearing children showed the positive effect of early appropriate intervention on phonologic skills development in hearing-impaired children. Phonologic skills support the ability of analyzing words into their phonologic elements and reading/ writing development in school. Moreover, control of produced speech pitch-loudness-rate and perception of heard speech pitch-loudness-rate results from phonologic skills development. Unsuitable usage of phonemes, word onset/offset consonant omission and a known vowel addition between two neighboring vowels are common phonologic disorders in hearing-impaired children that decreases the clarity of produced speech, especially in complex conversation backgrounds. Stress disorders result from inappropriate usage of phonetic duration, breathing control weakness, repetitious pauses in the speech continuum, breathing-speech producing imbalance, speech tonality disorder, and abnormal speech rhythm and enumerate as the most common speech abnormalities in hearing-impaired children. Identification/ intervention of hearing loss before the age of 6 months provides the possibility of language acquisition in hearing-impaired children in the same way as same as normal-hearing children, and reduces lingual abnormalities in these children (25).

Another finding of this study was a significant difference between the mean standardized scores of the combined lingual gains in the two assessed groups, which showed that children with early identified/intervened hearing loss have more prominent combined lingual gains than late identified/intervened children. The applied lingual gains were visual vocabulary, grammatical completion, word differentiation, word production, and phonologic analysis aspects. Lingual gains are predictive indicators for a child’s reading and writing abilities at school (26). Therefore, it is expected that children with late identification/intervention of hearing loss would have more reading and writing disorders and greater academic weakness in comparison with early identified/ intervened cases. Language development delay directly results from hearing loss and indirectly affects reading and writing abilities and mathematics learning. Hence, hearing-impaired children have lower social, academic, and educational success compared with their normal-hearing counterparts. It must be noted that academic improvement in hearing-impaired children also depends on their parents' co-operation, the quality/quantity of the intervention program, and the available supporting services (25).

No significant difference was seen between girls and boys in the lingual abilities assessed, showing that there is no effect of gender on language development of hearing-impaired children if they receive appropriate intervention. Hence, it seems that the effects of gender on improvement in lingual ability are seen only at the beginning of language acquisition (27).

Our findings were also consistent with those of Yoshinaga-Itano (2003) who showed that early identification/ intervention of hearing loss before the age of 6 months enables normal lingual/ cognitive development in hearing-impaired children regardless of their degree of hearing loss, gender, race, socioeconomic level and communicative methods. His study also revealed that children with early identified/intervened hearing loss have higher expressive language scores (28). In 1998, Yoshinaga-Itano noted that the mean length of speech in children with early identified/intervened hearing impairment is greater than that in late identified/ intervened children. The children with early identified/ intervened hearing impairment also use more vowels, consonants, morphemes and words intheir conversations than their late counterparts (29).

Based on the results of our study, the importance of early identification/ intervention of hearing loss is supported. Recent technology has made it easier to identify/ intervene in hearing loss at a younger age. Earlier studies have shown that the mean age of hearing-loss intervention in Persian hearing-impaired children was 3–6 years and found it has more recently been reduced to 2.5 years (26). Now, the technology has made it easier to identify/ intervention the hearing loss at younger ages. Upon earlier studies, the mean age of hearing loss intervention in Persian hearing impaired children was 3-6 years and found it is reduced to 2.5 years recently.

Because the relationship between lingual disorders and reading/writing disabilities is evident (30), it is possible to conclude that reading/writing and verbal language are connected modalities. Therefore, the perception and processing of reading/ writing language is closely related to verbal language. Hence, it is expected that children with early identified/intervened hearing impairment would have higher reading/writing abilities. However, as the children participating in this study were illiterate, it was impossible to study the relationship between the development of the lingual gains and reading/writing skills. 

## Conclusion

The present study supports the proficiency of the Persian TOLD-P3 test for perceptive/expressive language evaluation in Persian children with severe hearing impairment. Moreover, there was a significant difference in the synthetic and syntax skills of severely hearing-impaired Persian children with early versus late identification/intervention.Because synthetic and syntax skills are the basis for academic progress in school, so assessment of lingual development in hearing-impaired children will help in assessing lingual deficiencies and planning adequate auditory/verbal rehabilitative provision prior to entering school. Therefore, it is necessary to reduce the age of identification/ intervention of hearing impairment to 6 months or earlier in order to promote normal lingual development and proper emotional, academic, social and sensory growth.
